# Placenta-Like Structure of the Aphid Endoparasitic Wasp *Aphidius ervi*: A Strategy of Optimal Resources Acquisition

**DOI:** 10.1371/journal.pone.0018847

**Published:** 2011-04-19

**Authors:** Ahmed Sabri, Thierry Hance, Pascal D. Leroy, Isabelle Frère, Eric Haubruge, Jacqueline Destain, Philippe Compère, Philippe Thonart

**Affiliations:** 1 Centre Wallon de Biologie Industrielle, University of Liege, Liege, Belgium; 2 Earth and Life Institute, Biodiversity Research Center, University of Louvain-la-Neuve, Louvain-la-Neuve, Belgium; 3 Department of Functional and Evolutionary Entomology, University of Liege Gembloux Agro-Bio Tech, Gembloux, Belgium; 4 Laboratoire de Morphologie Ultrastructurale, University of Liege, Liège, Belgium; Ghent Universit, Belgium

## Abstract

*Aphidius ervi* (Hymenoptera: Braconidae) is an entomophagous parasitoid known to be an effective parasitoid of several aphid species of economic importance. A reduction of its production cost during mass rearing for inundative release is needed to improve its use in biological control of pests. In these contexts, a careful analysis of its entire development phases within its host is needed. This paper shows that this parasitoid has some characteristics in its embryological development rather complex and different from most other reported insects, which can be phylogenetically very close. First, its yolkless egg allows a high fecundity of the female but force them to hatch from the egg shell rapidly to the host hemocoel. An early cellularisation allowing a rapid differentiation of a serosa membrane seems to confirm this hypothesis. The serosa wraps the developing embryo until the first instar larva stage and invades the host tissues by microvilli projections and form a placenta like structure able to divert host resources and allowing nutrition and respiration of embryo. Such interspecific invasion, at the cellular level, recalls mammal's trophoblasts that anchors maternal uterine wall and underlines the high adaptation of *A. ervi* to develop in the host body.

## Introduction

Parasitoids are entomophagous insects that are playing an important role in natural ecosystems and in the control of crop pests. However, until now, despite the advantages they offer in biological control, their use remains largely limited compared with that of chemical pesticides. This is probably due to the difficulty to their mass production, which still relies on the classical approach and requires complex infrastructures to produce hosts (insects and plants) [1], [2].

Parasitoid usage in biological control should be greatly enhanced if a wide variety of these species could be mass produced by *in vitro* culture [3]. This should promote inundative strategies and extend biological control application to gradually replace chemical pesticides [2], [3], [4], [5]. However, development of suitable conditions and artificial diets requires an overall and detailed understanding of their developmental processes and interactions with the host [6].

*Aphidius ervi* (Haliday) (Hymenoptera, Braconidae) is an efficient parasitoid of several economically important aphid species and is found almost everywhere in the world [7], [8]. In last decade, several laboratories have explored the possibilities of its mass production and achieved a limited success [4], [9], [10], [11].

Pennacchio and Strand (2006) had underlined some aspect of its very complex embryological development, which involves profound interactions with the host [12]. *A. ervi* presents some embryological features rather different from free living insects, which can be phylogenetically very close [13]. First of all, its egg is hydropic, yolkless and contains very few energetic reserves, except some lipoid globules [13], [14]. In consequence, the early egg development depends on how fast the embryo is able to uptake resources from its host without being killed by the immune systems. Particular adaptation should render that possible. For instance, a kind of venom is injected by the female during oviposition [15], [16]. It acts directly on the germarium of the host and induces an inhibition of ovarioles development and stops the current development of aphid first larvae already present inside the aphid host. As pointed out previously [17], [18], the parasitoid larvae need also to regulate the host development and consumes gently his body to avoid killing it too rapidly. For instance, because of yolkless eggs, embryos have developed many fascinating strategies to redirect nutrients from the host body in their favour [11], [15], [19], [20].

In free living insects, embryo develops by consuming the maternally derived nutrients accumulated in vitellins [21], [22]. These reserves of nutrients, currently named yolk, are deposited in oocytes during the vitellogenesis. The amount of yolk in eggs is generally related to the development patterns and its consumption gives embryo all needed nutrients to achieve its development. In Hymenoptera order, which includes many parasitic wasps, eggs can be large and yolky as in free living species and ectoparasitic ones or small and yolk poor as in the endoparasitic species [13], [23]. This variability of eggs contents requires inevitably structural and functional adaptations, especially in extraembryonic membranes that are implicated in many important embryonic processes such as yolk processing and consumption [24], [25].

Among embryo extraembryonic membranes, serosa represents an important structure that develops from polar body or from embryonic cells at the start of embryogenesis [26]. For many authors, serosa has mainly contributed to the spectacular radiation of insects in terrestrial habitats [27]. It plays several roles such as blastokinesis, protection from desiccation and infections. In some species, serosa is involved in cuticle production and can supplant the protection provided by the chorion. It is also reported that it is implicated in yolk catabolism and active transport of resulting metabolic waste (for review see [25]).

Our aims were to decipher the key developmental phases of the endoparasitic wasp *A. ervi* throughout the embryological and early larval stages. We hypothesize that the adult female of this parasitic wasp developed a cuckoo-like strategy killing the aphid offspring inside its host and recreating a placenta like structure able to divert host resources from its own ovogenesis and allowing nutrition and respiration of parasitoid larvae.

## Results

### Early cellularisation and hatching

**Figure 1:**
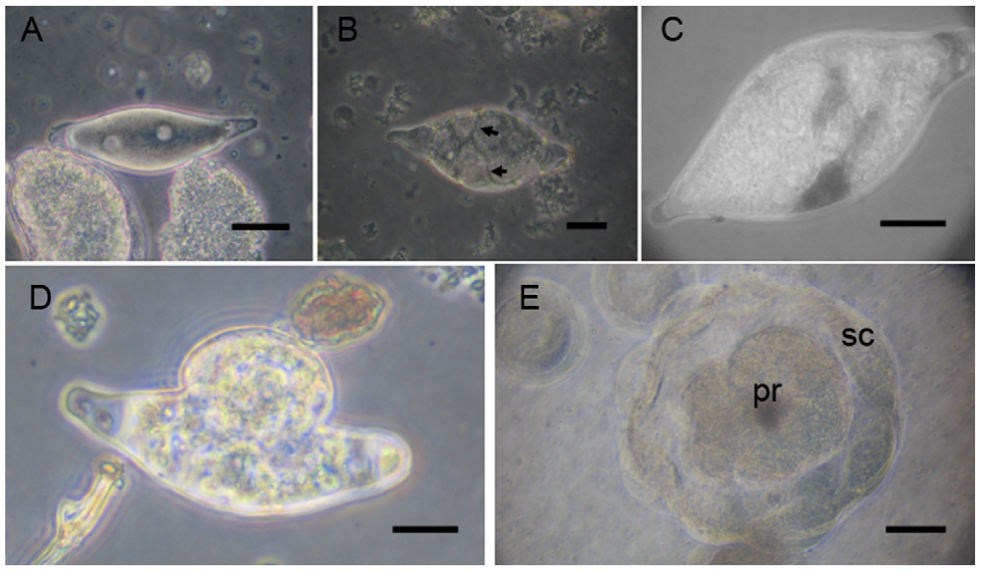
Egg development from oviposition to first hatching and release of a morula-like structure. **A**. Egg of *A. ervi* in the first hours after oviposition. **B**. Cellularisation occurs after limited syncytial cleavages, cell membranes are visible through the chorion (arrows in B). **C**. Folding processes of serosa cells beneath the egg chorion. **D**. Chorion rupture and embryo emergence from the chorion in the host hemocoel within the 24 h after oviposition (first hatching). **E**. Morula-like structure, about 24 h after hatching in the blastocoels. It is formed by the primordium (**pr**) surrounded by a monolayer of large cells, serosa cells (**sc**) forming the serosa membrane (Scale bars 25 µm for all panels). Abbreviations: pr, primordium; sc, serosa cells.

*A. ervi* egg reach 100±15 µm long with a very thin chorion (Figure 1A). It contains a weak amount of yolk compared to eggs of other free living Hymenoptera [13]. Following oviposition, the syncytial phase, characteristic of insect development is almost non-existent as already mentioned by Grbic (2000) [13]. Blastomers separated by cell membranes are detected through the chorion very soon after the first nuclear cleavage (Figure 1B). Then, two types of cells that differ in size become visible through the chorion. The largest ones (20±5 µm in diameter) are often just under the chorion and will produce the serosa membrane. The smallest cells (about 8±3 µm in diameter) constitute the primordium.

Early cellularisation followed by the folding processes of serosa cells (Figure 1C) occur within the chorion and causes a slight swelling of the egg. The chorion rupture occurs after 24 h (Figure 1D) and may be considered the true egg hatching. It produces a morula-like structure of about 150±40 µm of diameter bathing in the host haemocoel (Figure 1E) and composed of a cluster of embryonic cells surrounded by a monolayer of 16 cells forming the serosa membrane. The primordium lies in that structure surrounded by a blastocoel. It is thus well separated from the host hemocoel.

### Morula-like structure anchorage in the host fat body

**Figure 2:**
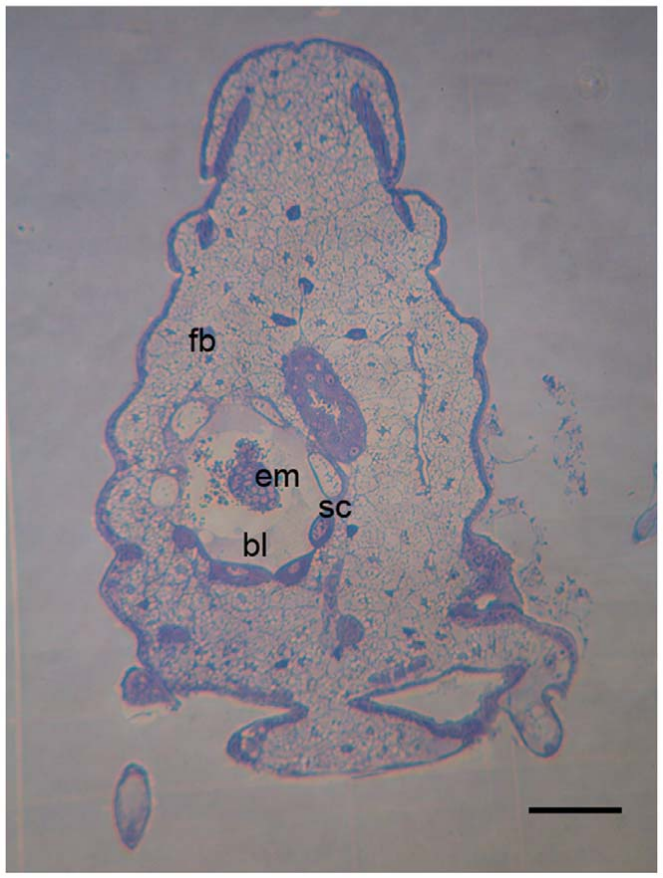
Semithin section of *A. pisum* parasitized by *A. ervi* embryo after about 48 hours of development. Parasitoid embryo begins its development surrounded by a serosa membrane (Scale bar 100 µm). Abbreviations: fb, fat body; em, embryo; bl, blastocoel; sc, serosa cells.

Following its liberation in the host hemocoel, the morula-like structure has still about 150 µm diameter and is clearly anchored in the host tissue, mainly in fat body (Figure 2). During the following 24 h, its size increases progressively to reach about 200 µm of diameter. Cells forming the serosa undergo limited divisions and give about 32 cells organized in a continuous monolayer of cells around the blastocoels, which bathes the developing embryo. Serosa cells appear completely separated from embryo that floats in the blastocoels.

### Embryogenesis of A. ervi and second hatching

**Figure 3:**
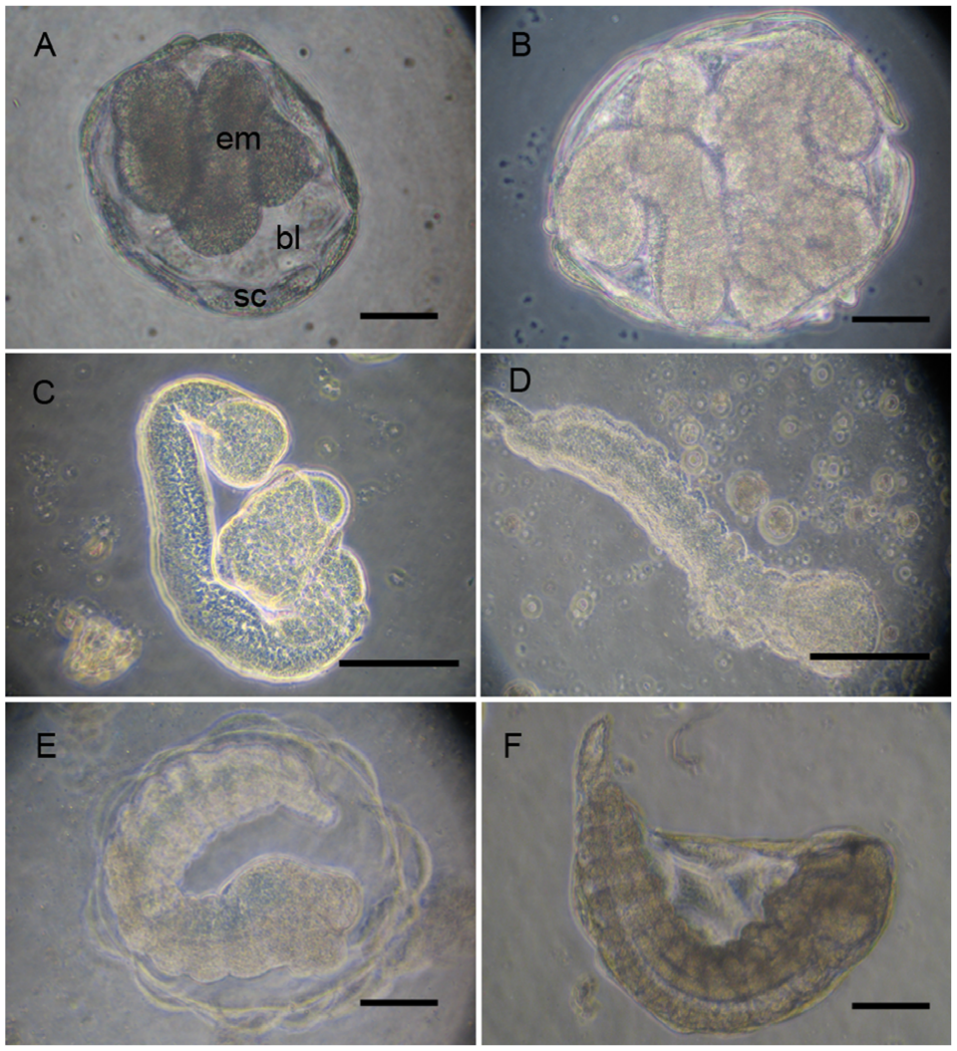
*A. ervi* embryo development from the first to the second hatching. Until the 5^th^ day of development, all embryonic and 1st instar larva stages occur within the blastocoels (**bl**) delimited by the serosa membrane (**sc**). **A,B**. The primordium undergoes its growth by extension of the posterior part and the embryo (**em**) acquires a coiled shape, about 70 h after oviposition. **C,D**. The elongation phase is followed by a condensation and segmentation phases as illustrated in **C** and **D**, where the serosa was removed. **E,F**. Embryo reaches its full development after about 120 h and a 1^st^ instar larva hatches in the aphid hemocoel by ripping off the serosa (second hatching). (Scale bars 100 µm for all panels). Abbreviations: bl, blastocoel; em, embryo; sc, serosa cells.

During embryogenesis, serosa membrane shelters the embryo which develops in the blastocoels. Just after hatching, the primordium consists of a cluster of cells and undergoes a germband elongation (Figures 3A, 3B). Seventy hours after oviposition, still within the blastocoels, the embryo acquires a coiled shape. A process of condensation and segmentation is then initiated in an anterior-to-posterior sequence (Figures 3C and 3D where serosa was removed). One hundred h after parasitization, metamerisation is clearly apparent through the serosa and the embryo is gradually transformed in a first instar larva (Figure 3E). Until the 5^th^ day of development, the embryo has no direct interaction with the aphid tissues and all nutrients exchanges are done through the blastocoels and through the serosa membrane. After about 120 h, the 1^st^ instar larva hatches in the aphid hemocoel by ripping off the serosa (Figure 3F).

### Serosa membrane ultrastructure

**Figure 4:**
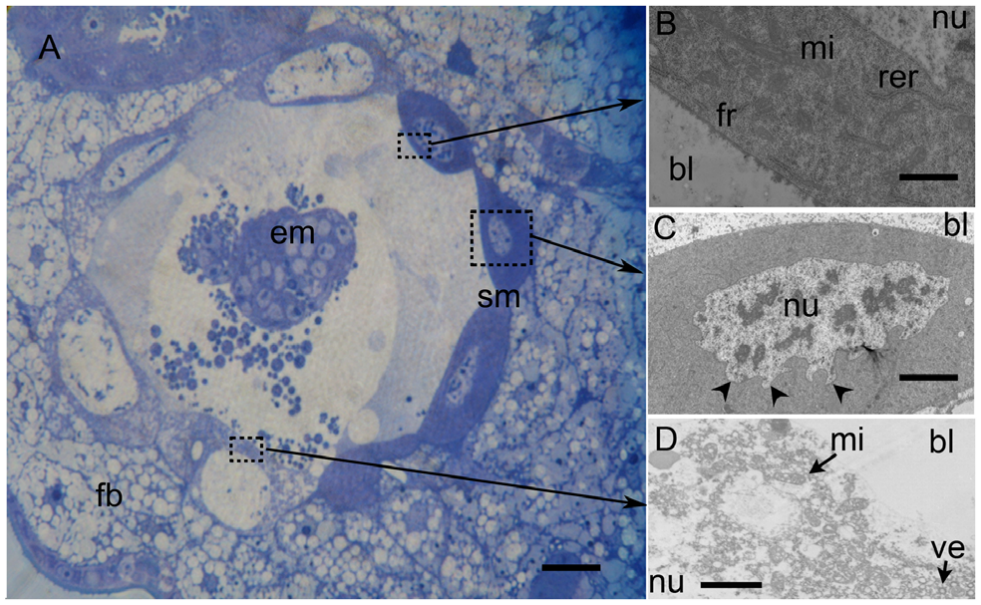
Semithin and thin sections of *A. ervi* embryo and its serosa membrane. **A**. 48 h old embryo (**em**) in *A. pisum,* the serosa membrane (**sm**) is anchored in the fat body (**fb**) of the host and is formed by two types of cells with different cytoplasm density. **B**. TEM micrograph revealing that cells with dense cytoplasm are very rich in free ribosomes (**fr**) and contain many mitochondria (**mi**) and rough endoplasmic reticulum (**rer**). **C**. The nuclear membranes of dense cells are irregular on the side facing aphid tissue (arrowheads in **C**). **D**. The second type of cells has less dense cytoplasm with relatively large nucleus (**nu**) (visible in panel A). Their cytoplasm contains many mitochondria and small vesicles (**ve**). Scale bars  = 25 µm in A, 1 µm in B, 10 µm in C and 2 µm in D. Abbreviations: em, embryo; fb, fat body; sm, serosa membrane; bl, blastocoel; fr, free ribosomes; mi, mitochondria; nu, nucleus; rer, endoplasmic reticulum; ve vesicles.

Light and transmission electron microscopy (TEM) analysis showed that the serosa membrane is composed of two kinds of large cells showing differences in their cytoplasm density (Figure 4A).

Cells with dense cytoplasm show a very high density of free ribosomes and contain many rough endoplasmic reticulum and mitochondria (Figure 4B). Their nuclei are irregular at the side facing the apical membrane of cells (Figure 4C). Cells with less dense cytoplasm have large nuclei and their cytoplasm contains many mitochondria and small vesicles (Figure 4D).

**Figure 5:**
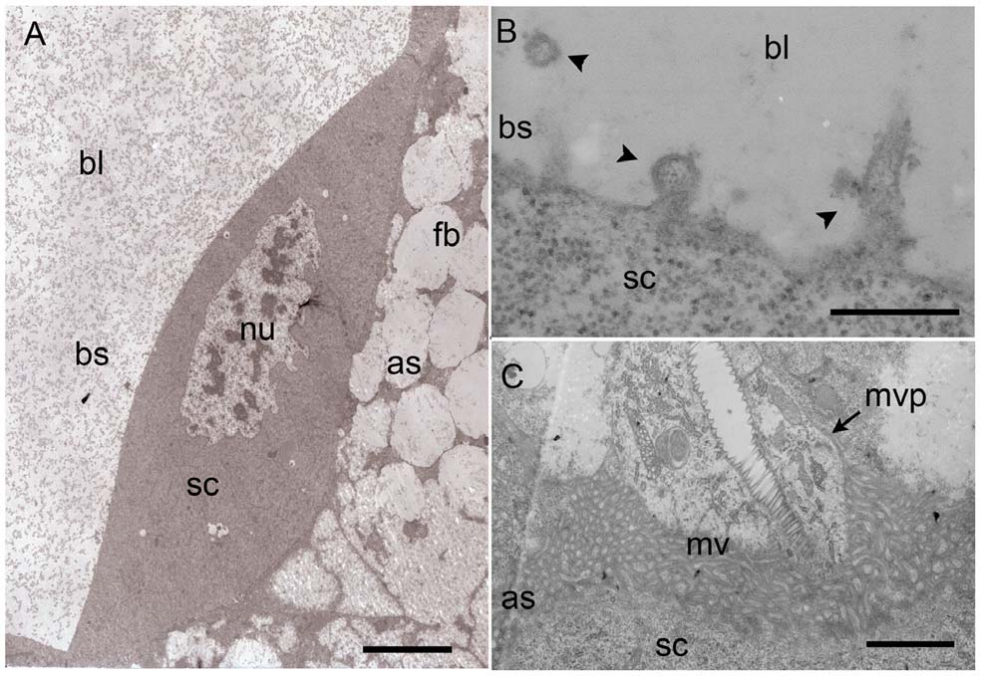
TEM micrographs of the serosa cells 48 h after oviposition. **A**. Serosa cells (**sc**) are highly polarized with separate apical and basal membrane. **B**. The basal side (**bs**), facing the blastocoel (**bl**) shows several exocytosis vesicles (**ve**) (arrowheads in **B**). **C**. The apical side (**as**), facing the parasitized aphid tissue, shows microvilli (**mv**) and microvilli projections (**mvp**) in the aphid tissue. Scale bars  = 10 µm in A, 1 µm in B and 3 µm in C. Abbreviations: as, apical side; bl, blastocoel; bs, basal side; fb, fat body; mvp, microvilli projections; mv, microvilli; nu, nucleus; sc, serosa cells.

For both kinds of cell, we found that plasma membrane has domains with profound morphological differences (Figure 5). Their basal side (facing the embryo) appears regular and uninterrupted. However, magnifications reveal the formation of several vesicles, which are released in the blastocoel corresponding probably to an exocytose of nutrients (Figure 5B). The apical side (host body oriented side) is crowded with microvilli, which extend into the host fat body and allow the anchorage of the serosa membrane in the parasitized aphid tissue (Figure 5C).

**Figure 6:**
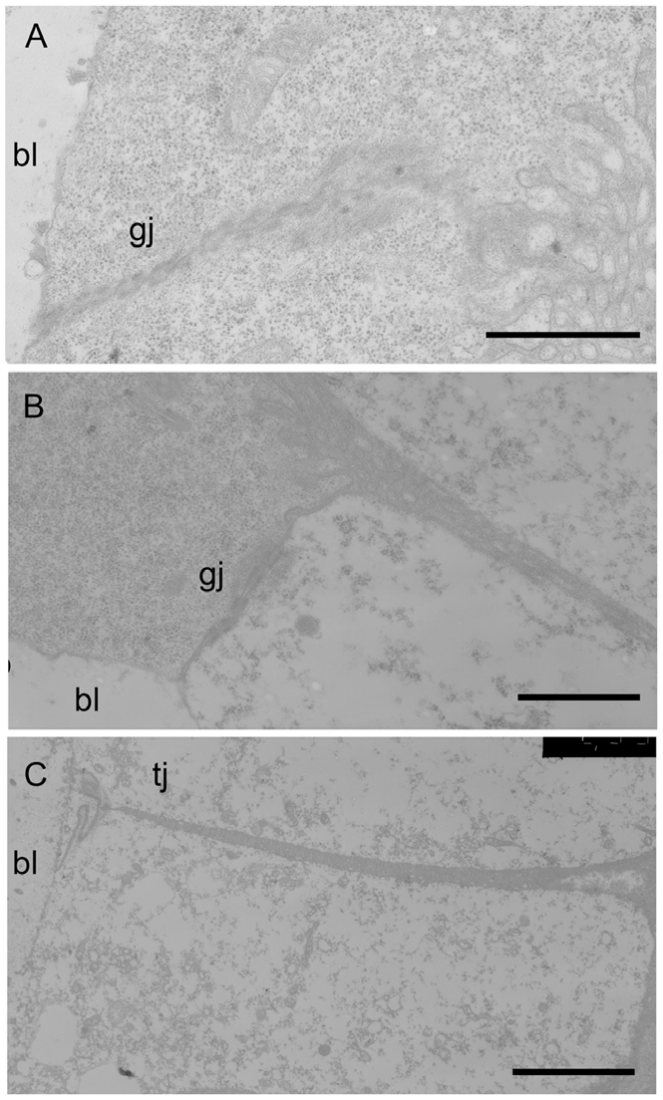
Junctions observed between serosa cells after 48 h of parasitoid development. **A,B**. Gap junctions (**gj**) are mainly present between cells with dense cytoplasm and between dense cells and cells with less dense cytoplasm fraction. **C**. Tight junctions (**tj**) are observed between cells with less dense cytoplasm. **bl**, blastocoel. Scale bars  = 5 µm for all panels. Abbreviations: bl, blastocoel; gj, gap junctions; tj, tight junctions.

Serosa cells are organized in a continuous monolayer and provide an interface between host tissues and the blastocoel bathing the embryo. Two types of junction are observed between serosa cells, gap junctions (gj) are mainly observed between adjacent cells with dense cytoplasm (Figure 6A). The same junctions are also observed between cells with dense cytoplasm and cells with less dense cytoplasm (Figure 6B). The second types of junctions are tight junctions and seal spaces between adjacent less dense serosa cells in a narrow band beneath their basal surface (facing the blastocoels) (Figure 6C).

**Figure 7:**
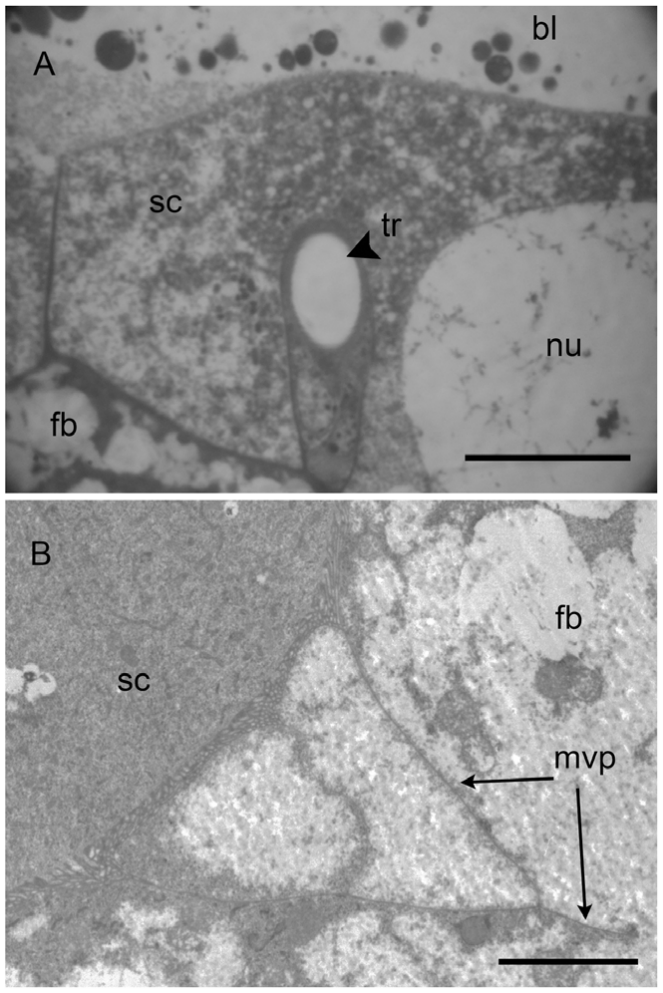
The serosa is anchored in the fat body of parasitized aphid. **A.** Some serosa cells (**sc**) grow around the host trachea (**tr**) (arrowhead in **A**). **B**. The apical side of some other serosa cells forms microvilli projections (**mvp**) that extend deeply into the host fat body (**fb**). Scale bars  = 10 µm in A and 5 µm in B. Abbreviations: bl, blastocoel; fb, fat body; mvp, microvilli projections; nu, nucleus; tr, trachea; sc, serosa cells.

High magnification confirmed that serosa is anchored in the host tissues. Figure 7A reveals that some cells of the serosa grow around the host trachea providing oxygen to the developing embryo. Many other serosa cells show microvilli projections, at their apical side, extended deeply into the host tissues (frequently up to 20 µm) (Figure 7B). This lead to think that they are acting like a digestive epithelium.

### Serosa membrane dissociation and teratocytes release

**Figure 8:**
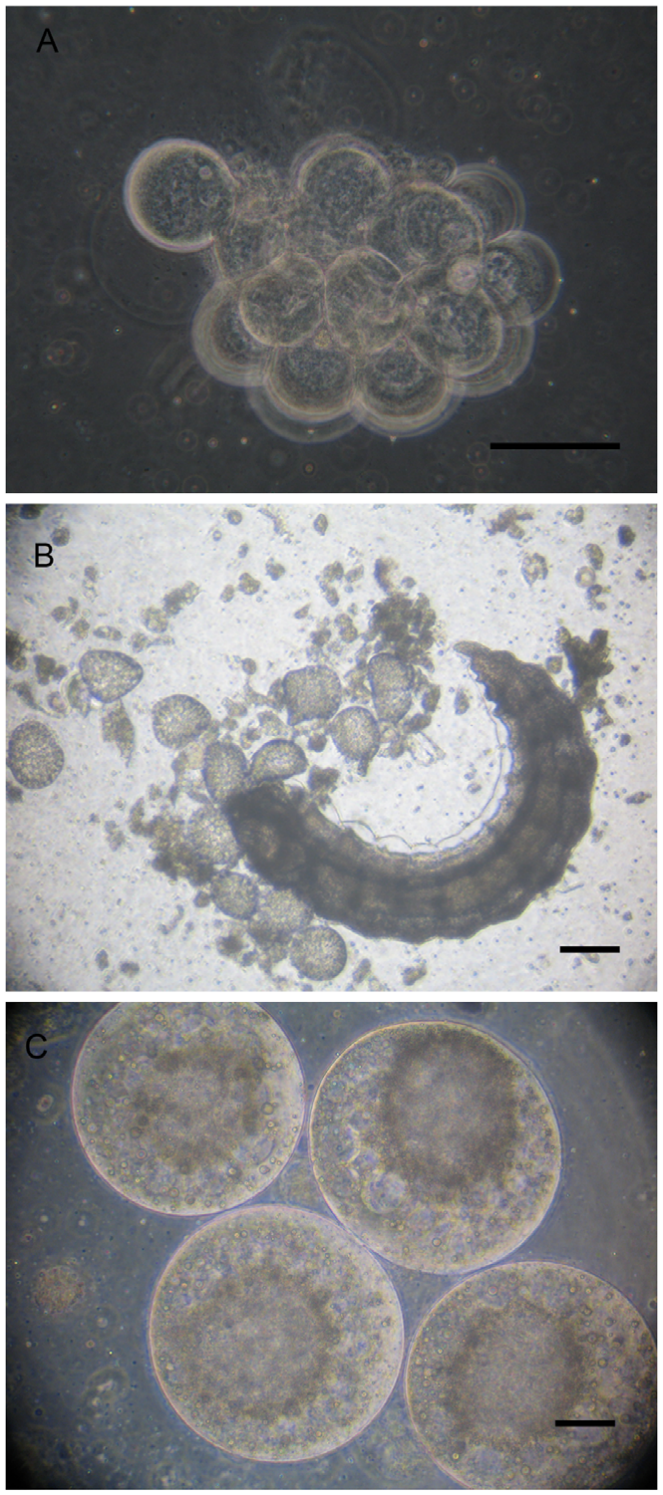
Serosa dissociation into teratocytes. **A, B**. Serosa cells dissociate to free teratocytes in the aphid hemocoel after the larva hatching. **C**. Teratocytes sizes reach about 200±50 µm (about 5 fold their size at the hatching). Scale bars  = 50 µm.

Cells of serosa membrane dissociate into free teratocytes as soon as the parasitoid larva egresses in the host haemocoel (Figures 8A, 8B). Five days after oviposition, about 30±5 free teratocytes were observed in the host. After their release, their number remains almost the same up to 7 days after parasitization and then decreases drastically to about 18±5 and 2±1 teratocytes respectively on days 8^th^ and 9^th^. Their size at their release is about 40±10 µm diameter and increase during the two following days up to approximately 200±50 µm. The cytoplasm fraction of many of them became progressively crowded with vesicles, which appear to be similar to lipid droplets of the host fat body cells (Figure 8C).

## Discussion

It is often reported that nutritional needs of parasitoids are not different from those of other free-living insect species. However, nutritional investigations focused principally on dietary and nutritional requirements have achieved a limited success yet, especially for the endoparasitic ones [28]. For the parasitoid *A. ervi*, it is increasingly clear that this failure is not only due to inappropriate culture media but is also due to other factors such as certain particularities of its embryological development.

Parasitoid wasps have evolved a wide spectrum of developmental strategies in response to parasitic life [12]. *A. ervi* seems in agreement with this and shows a rather complex embryological development that is different from many other species which can be phylogenetically very close. Indeed, nearly all insect embryos undergo a syncytial phase of development during the initial cleavages of embryogenesis and consists of nuclear mitotic divisions without cell membranes formation [29], [30]. In the case of *A. ervi,* early embryogenesis seems to lack this universal phase and cell membranes are distinguishable through the chorion simultaneously with the first divisions. Grbic (1998) has demonstrated using intracellular tracer dye injections that this phase, in *A. ervi,* is so short that only the first cleavage occurs in a syncytium [31]. The same author suggests that early cellularization is not an exception in parasitic wasps and can be related to the mode of nutrition and the amount of yolk in their eggs. For example, the endoparasitic wasp *Copidosoma floridanum* (Ashmead) (Hymenoptera, Encyrtidae) has a small and yolk-poor egg and undergoes an early cellularisation similar to that for *A. ervi*. In contrast, the ectoparasitic wasp *Bracon hebetor,* despite that it is phylogenetically most close to *A. ervi,* its large and yolky egg undergoes long germband development including 12 syncytial nuclear divisions before cellularization [13], [32].

The lack of syncytial phase and early cellularisation in *A. ervi* egg are correlated with a rapid differentiation of large cells that give the serosa membrane upon hatching. If one considers these observations and the low content of yolk in the egg, it is tempting to say that the embryo seems in a “time trial” to leave the eggshell as soon as possible to uptake its nutrients from host hemocoel with an appropriate structure without any barriers. Furthermore, this hypothesis is supported by premature emergence of the morula-like structure from the egg chorion, within 24 h.

For other parasitoid Hymenoptera, close to *A. ervi*, hatching from the chorion occurs much later and the released larvae are at advanced stages of their development. This is the case for *Cardiochiles nigriceps* (Viereck) (Hymenoptera: Braconidae) where egg volume increases and stretches the chorion several hundredfolds [33], [34]. *Microplitis croceipes* (Cresson) (Hymenoptera: Braconidae) hatches from the chorion about 40 h after oviposition with the aid of abdominal contractions of first instar larva [35]. *Cotesia congregata* (Say) (Hymenoptera: Braconidae) and *Meteorus pulchricornis* (Wesmael) (Hymenoptera: Braconidae) emerge from the chorion 48 to 72 h after oviposition [36], [37]. Therefore, for all cited cases, the chorion remains around the embryo until firsts instar larvae stages and its rupture release larvae and free teratocytes in the host body. For *A. ervi*, chorion rupture happens too early, but the released primordium remains readily wrapped in the serosa membrane until the 5^th^ day of its development.

An overview of the literature shows that extraembryonic membranes are regarded as an important aspect of insect embryogenesis and represent one of insect's evolutionary innovations that contribute to their high adaptability to environmental conditions and their ability to conquer various ecological niches [25], [27], [38], [39]. Most insects develop two extraembryonic epithelia from an amnioserosal fold, called amnion and serosa. The serosa detaches from the amnion and encloses the embryo amnion and yolk [25], [38], [39], [40], [41], [42], [43].

For endoparasitic species of Hymenoptera, the serosa has been studied in relatively few species and most available information concerns its dissociation after hatching to teratocytes [34], [36], [44], [45], [46].

So, even if *A. ervi* represents one of the most studied endoparasitic hymenoptera and although its embryonic development has received a great interest in the last decades there is no information about the serosa. Yet the latter seems to be devoted to play a key role in the control of all embryo interactions with the host since until the emergence of a first instar larva, it represents an interrupted interface between embryo and the host body. A nutritional role of the serosa has been already reported by Pennacchio et al. 1994 for *Cardiochiles nigriceps* which, as for *A. ervi*, the embryo remain wrapped by different types of cells forming a continuous serosa [34]. Exocytosis-vesicles at the basal side of cells as well as their abundant mitochondria, free ribosomes and rough endoplasmic reticulum reflect a high metabolic activity and suggest an active role in nutrient processing before their release into the blastocoel. Such characteristic seems shared by serosa cells of many other reported endoparasitic hymenoptera species [36], [45], [46]. Moreover, it has been demonstrated that for *Microplitis croceipes*, the major contribution of the female to its progeny development consists in furnishing a great number of ribosomes and mitochondria, which allow an active protein synthesis during embryogenesis. All proteinic requirements are taken up from the host hemolymph as free amino acids [14], [47], [48], [49]. On the other hand, the serosa of most reported species seems to contain different types of cells with deep cytological differences suggesting that serosa role is very complex and is certainly not limited neither to a nutritional nor a protective roles.

Serosa polarity has been described by Grimaldi et al. (2006) for the tobacco budworm parasitoid *Toxoneuron nigriceps* [45]. In this case, the serosa membrane is formed by a layer of cells showing microvilli both in the basal and apical side. At the apical side, microvilli are organizedin a brush border and covered by a thick amorphous felt. Conversely, the serosa of *A. ervi* appears deeply anchored in the aphid body without a clear margin or any protective structure. The serosa implantation in the host body is confirmed by cellular expansion around host trachea and microvilli projections in neighbors' tissues. To our knowledge, such invasion of host tissue by the serosa cells represents a particularity never reported previously for other parasitoid and recalls mammal's trophoblasts that allow embryo implantation to maternal uterus.

On the other hand, back on the topic of the *invitro* culture of this valuable insect, it is important to note that in animal cell culture most cells need a surface or substrate to adhere to so that they are able to develop [50], [51], [52]. This leads, therefore, to believe that *A. ervi* serosa anchorage in host tissues could be among the causes of the limited success of in vitro culture and represents a requirement that needs also to be taken into account to improve our chances to produce it.

In conclusion, *Aphidius ervi* have developed an original way of development to optimize host resources exploitation. The eggs' weak content of yolk allows female to reduce its investment in the vitellogenesis and to have a high fecundity [7], [14], [23]. Moreover, the host embryos of the aphids already in development are killed by the mother venom injection allowing a reallocation of all host resources to the parasitoid [15], [16], [53]. This recalls the cuckoo strategy wherein the female reduces its investment in its eggs and progeny making the host carry the burden of nurturing the offsprings. The lack of yolk in the egg is overcome by a very rapid hatching of the embryo at its earliest stage of development as a cluster of primordial cells wrapped in the serosa membrane. The latter membrane clearly acts like a mammal's placenta pumping the resources and oxygen from host tissue and in view of our observations it is tempting to say that among all the fascinating strategies adopted by *A. ervi*; those concerning the serosa deserve more than ever a special attention since they clearly make it on the “front line” of the host body conquest.

## Materials and Methods

### Rearing conditions

The parasitoid *A. ervi* was reared on *A. pisum*, maintained at least one week on old seedlings of *Vicia faba* (broad bean). Aphid and parasitoid cultures were kept in separate rearing chambers under 20°C, 75% RH and 16 h L: 8 h D photoperiod. *A. pisum* cultures were started in 1999, with insects collected by the Department of Functional and Evolutionary Entomology from Lucerne plants, in Belgium. *A. ervi* was provide by Viridaxis SA (Belgium).

### Host parasitization and dissection

Synchronised larvae (4 day old) of the host aphid *Acyrthosiphon pisum* were produced by leaving pea aphid adults to reproduce on broad bean plants for 8–10 h. The adults were then removed and the progeny produced over this time interval was maintained on the same plants under 20°C and 16 h L: 8 h D photoperiod. Four days old aphids were parasitized by *A. ervi* females in a petri dish (Ø×10 cm). After parasitization, aphids were placed back to broad bean plants and kept under the same environmental conditions until dissection. To examine the parasitoid development, at different time intervals after parasitoid oviposition, parasitized aphids were anaesthetised with CO_2_ then carefully dissected in a petri dish in 100 µl of Shield and Sang medium (Sigma). A stereo microscope Wild M3B was used for dissections and selected samples were immediately transferred to an inverted microscope Olympus CK40 to take photographs. After parasitization, every 24 h, 40 to 50 parasitized aphids were processed.

### Histological analyses

Semithin and thin sections were performed. Parasitized aphids were fixed by direct immersion for 3 h at room temperature in a 2.5% glutaraldehyde solution buffered with 0.2 M Na-cacodylate at pH 7.4. The osmolarity was adjusted to 850 mOsm by addition of sucrose (5%). All samples were post-fixed in glutaraldehyde for 2 h at 4°C in buffered 1% OsO 4, rinsed in distilled water, dehydrated in an ethanol-propylene oxide series, and embedded in epoxide (Glycidether 100, Serva). Flat silicone rubber moulds were used to facilitate orientation before sectioning. Aphids were cut into several semithin sections (1 µm thick) using glass knives (Ultramicrotome LKB or Reichert-Jung Ultracut E). Sections were then stained with toluidine blue for light microscopy in 1% toluidine blue at pH 9.0 before observation under an Olympus microscope. Selected samples were cut into ultrathin sections for transmission electron microscopy with a diamond knife and contrasted with uranyl acetate and lead citrate before examination with a JEOL TEM (JEM 100-SX) at 80 kV accelerating voltage.
